# Anorectal metastasis from breast carcinoma: a case report and review of the literature

**DOI:** 10.1186/s13104-018-3356-z

**Published:** 2018-05-02

**Authors:** Hannes Ruymbeke, Luc Harlet, Barbara Stragier, Edwin Steenkiste, Merijn Ryckx, Francois Marolleau

**Affiliations:** 1 Resident in Internal Medicine, Ghent University, Ghent, Belgium; 2 Breedstraat 265, 9100 Sint-Niklaas, Belgium; 3 Department of Gastroenterology, AZ Delta, Menen, Roeselare, Belgium; 4 Department of Medical Oncology, AZ Delta, Menen, Roeselare, Belgium; 5 Department of Anatomopathology, AZ Delta, Menen, Roeselare, Belgium

**Keywords:** Breast cancer, Metastasis, Gastrointestinal, Anorectal

## Abstract

**Background:**

Gastrointestinal metastasis from primary breast carcinoma is uncommon, anorectal involvement is extremely rare.

**Case presentation:**

We present the case of a 65-year old woman who underwent treatment for an infiltrative lobular carcinoma of the left breast with bone metastases and who developed metastasis of the rectum and anal canal 4 years later.

**Conclusions:**

A patient with a history of breast cancer, especially lobular carcinoma, presenting with anorectal symptoms, should raise the suspicion of metastatic disease.

## Background

Breast cancer (BC) is the most prevalent cancer in females, affecting one in eight women in their lifetime [1, 2]. Although mortality rates are decreasing in western countries, worldwide it is still the leading cause of cancer death among females [3]. Invasive lobular carcinoma (ILC) is the second most common type of invasive breast cancer, following ductal adenocarcinoma. ILC accounts for approximately 5–15% of the invasive breast carcinomas and its incidence is increasing. Patients with BC have metastatic disease in around 50%. BC is known to spread to the bones, lungs, liver, brain and soft tissues. However, gastrointestinal (GI) metastasis is rare in clinical practice. It occurs only in less than 1% of all metastatic cases and the metastatic rate is higher for ILC than for ductal adenocarcinoma. In the GI tract, the stomach is the most commonly affected organ, metastatic spread to the rectum and anal region is extremely rare [4–6].

A thorough review of literature revealed only six individual cases of anal involvement so far (Table 1) [7–12].

**Table 1:** Reported cases of anal metastasis from breast carcinoma

Case	Age (years)	Histology	Interval	Clinical presentation	Therapy	Survival
Dawson et al. [7]	70	ILC	34 months	Altering bowel habit, constipation, anal discharge	Laparotomy and RT	?
Haberstich et al. [8]	78	IDC	At diagnosis	Painful anal tumefaction and blood loss with stools	Abdominoperineal resection and RT	Disease-free at 22 months follow-up
Nair et al. [9]	57	IDC	7 years	Alternating bowel habit, crampy lower abdominal pain, increased frequency of bowel movements	Colostomy and RT	?
Puglisi et al. [10]	92	ILC	4 years	Tenesmus and painful anal polypoid lesion	RT and hormonal therapy	3 years
Bochicchio et al. [11]	72	ILC	4 years	Constipation, tenesmus, fecal incontinence	Hartmann rectal amputation and RT	Few months after RT
Rengifo et al. [12]	78	IDC	27 months before diagnosis of BC	Rectal bleeding, weight loss, constipation	RT and hormonal therapy	?

*ILC* invasive lobular carcinoma, *IDC* invasive ductal carcinoma, *HT* hormonal therapy, *RT* radiotherapy

## Case presentation

In May 2016, a 65-year old woman presented at the Department of Gastroenterology with a 1-month history of altering pattern of stool, intermittent fecal incontinence and tenesmus. There was no history of rectal bleeding. Digital rectal examination revealed a hard invasion of the anal canal.

In 2012 she had been diagnosed with a left-sided invasive lobular breast carcinoma with extensive bone metastases. Immunohistochemical staining was positive for estrogen receptor (ER) and progesterone receptor (PR) and negative for human epidermal growth factor receptor 2 (HER2-neu). The tumor cells showed a low mitotic index (5%). She was treated with denosumab and hormonal therapy: first with tamoxifen, which was later on replaced by letrozole and eventually by exemestane due to progressive bone metastases. Because of progression of the primary tumor and the fear of future wound ulceration, the patient decided to undergo a left-sided mastectomy and prophylactic contralateral mastectomy in 2013.

**Fig. 1:**
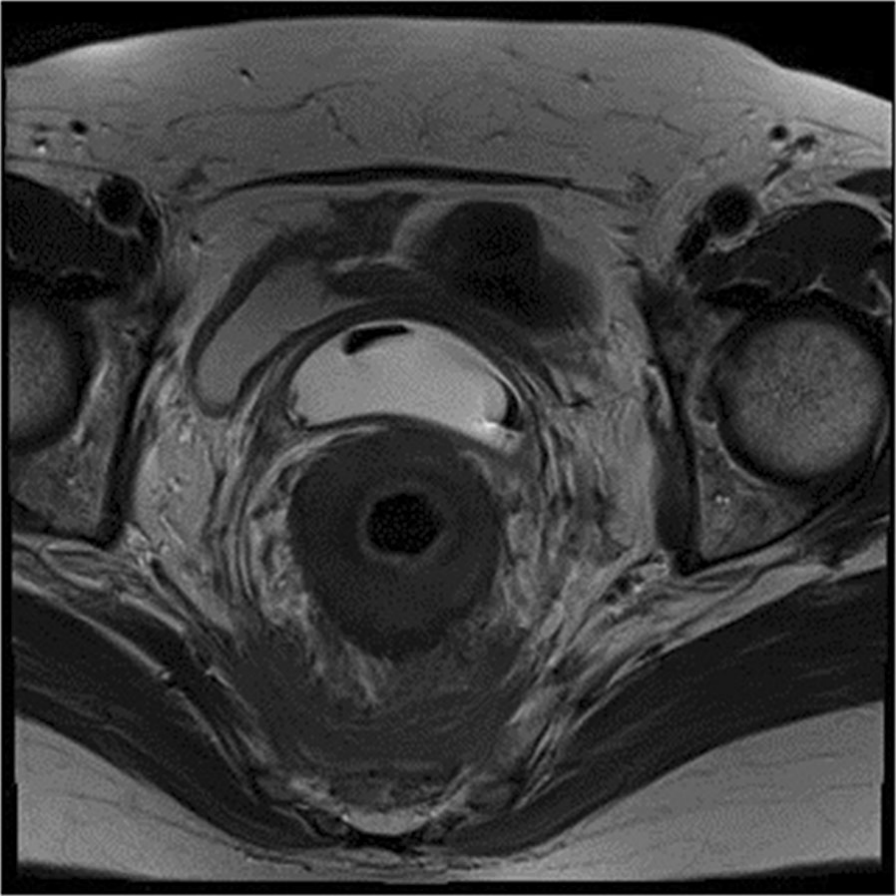
MRI shows diffuse wall thickening of the rectum and infiltration of the presacral fat tissue

**Fig. 2:**
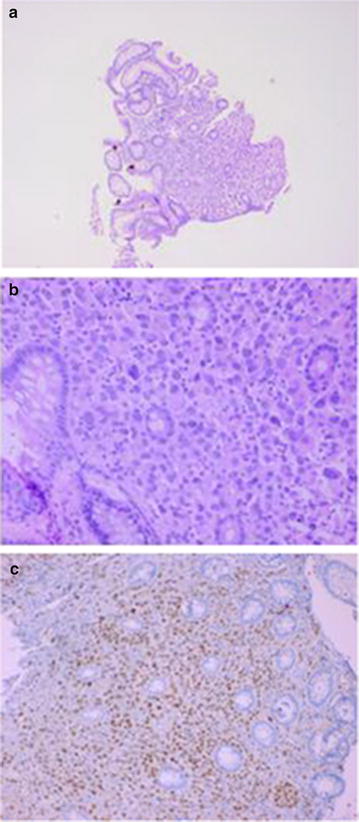
Cytomorphological and immunohistochemical features of a specimen from a lobular breast carcinoma metastasis in the anorectal lesion, obtained through endoscopy and biopsy. **a**, **b** Hematoxylin and eosin staining at various magnifications, ×5 and ×20 respectively. **c** Estrogen receptor positivity, magnification ×10

At the initial presentation in May 2016, complete blood count, renal and liver function tests were normal, C-reactive protein was not elevated. Cancer antigen (CA) 15.3 was elevated (98 kU/l vs. 61 kU/l 8 months earlier, ref. < 30), there was a normal value of carcinoembryogenic antigen and CA 125. Left colonoscopy showed narrowing of the anal canal due to an anorectal mass and biopsies were taken. Abdominal computerized tomography (CT) revealed thickening of the anorectal wall and the soft tissues presacral and showed a few small lymph nodes in the anterior perirectal fat tissue. Magnetic resonance imaging (MRI) scan revealed diffuse wall thickening of the rectum and anal canal and infiltration of the presacral fat tissue and rectovaginal septum, more suggestive of inflammatory changes than of tumoral pathology (Fig. 1). Except for bone metastases, no other metastatic lesions were identified on CT scan of the thorax and the abdomen. Anatomopathology revealed that the biopsies were consistent with metastatic rectal lesions of a primary lobular breast carcinoma. The immunohistochemical staining was similar to the primary breast tumor (Fig. 2).

Before radiotherapy could be started for this new metastasis, the patient developed colonic obstruction due to the anorectal tumor, so a colostomy had to be created laparoscopically. During laparoscopy, a tumoral implant was found on the abdominal wall, later confirmed to be a metastatic lesion as well. Afterwards local radiotherapy was given to a total radiation dose of 39 Gy in 13 sessions of 3 Gy. Five months later there was a strong increase in CA 15.3 value. New imaging revealed progressive bone metastases and a thickened stomach wall. Gastroduodenoscopy confirmed an infiltrated stomach wall at the greater curvature, but biopsies could not confirm malignancy. Everolimus, an oral mTOR-inhibitor, was associated with exemestan. Another 5 months later the patient developed ascites. Medical imaging showed mesenterial metastases and CA 15.3 value kept on increasing. Hormonal therapy was stopped and chemotherapy was initiated (6 cycles of epirubicin + cyclophosphamide), which led to an important decrease in CA 15.3 value (340–29 kU/l). She now remains alive 2 months after ending chemotherapy and 15 months after the diagnosis of anorectal metastasis.

## Discussion and conclusion

Breast cancer is the second most common cancer to metastasize to the GI tract, following malignant melanoma. Although GI metastasis from invasive breast cancer is rare (< 1% in clinical practice), the incidence might be underestimated and is likely to increase [7, 8]. Autopsy series report a rate of GI tract metastasis up to 15% [8]. With early diagnosis and as treatment regimens improve, survival rates of breast cancer are increasing. With an ageing population and an increasing number of cancer survivors we might expect to encounter more unusual presentations with distant metastasis in the near future [9, 10, 12].

ILC has a metastatic rate of 4.5% to the GI tract, invasive ductal carcinoma only 0.2% [8]. GI metastasis is usually associated with extensive systemic spread [8]. Sites of metastasis can vary from the oropharynx to the anus. Anorectal involvement is very rare [13, 14]. Rectal metastasis from ILC usually occurs 5–7 years after diagnosis of the primary tumor, but there have been cases reported with synchronously rectal metastasis, as well as metastasis up to 20–30 years after diagnosis of primary ILC [5, 15].

A thorough review of literature was performed in PubMed, Embase and Web of Science. We used the following search terms: ‘Breast Cancer’ in combination with ‘Anal Metastasis’, ‘Anorectal’ and ‘Anal Canal’. This search method revealed only six individual cases of anal involvement so far, three with a history of invasive lobular carcinoma and three with a history of invasive ductal carcinoma (Table 1) [7–12].

The combination of its rarity, the often unusually long interval and the non-specific clinical presentation makes the diagnosis of GI metastasis from primary breast carcinoma a challenge. Early recognition and correct diagnosis is important for an adequate therapeutic strategy [9, 14].

GI metastatic disease should always be considered when a patient with a history of BC presents with abdominal complaints, even many years after the diagnosis of BC. Symptoms depend on the localization of metastatic disease, such as diarrhea, constipation, obstruction, incontinence, weight loss, tenesmus. Most symptoms are non-specific and may be considered treatment-related or as inflammatory GI disease. They can also mimic a primary cancer of the GI tract, which is more common than isolated GI metastasis of primary BC [2, 5].

Imaging studies may be helpful for diagnosis, but radiological features may mimic primary neoplasms or inflammatory changes, as was the case for this patient.

Endoscopy is important in making a correct diagnosis [8, 14]. The metastatic lesion may simulate inflammatory bowel disease macroscopically, so taking biopsies is important. The most common manifestation of anorectal involvement is diffuse infiltration, leading to thickening and rigidity of the rectal wall and narrowing of the lumen. As is the case for our patient, most rectal lesions appear more linitis plastica-like, rather than a solitary intraluminal mass [15]. Although endoscopy with biopsy remains the best diagnostic method, it can give false negative results, for example when the tumor is submucosal. Biopsies of the thickened stomach wall of our patient could not confirm metastatic disease, although it was a suspect lesion macroscopically. Repeated biopsies may be necessary [13–15].

Histopathological diagnosis of metastatic lesions can be challenging as well. The lack of dysplasia of the rectal mucosa is often helpful in distinguishing between a primary and metastatic lesion. Immunohistochemical techniques will allow for the most accurate diagnosis. ER, PR and HER2-neu status should be compared with the features of the primary breast tumor [4, 16].

Data on treatment for patients with BC and GI metastasis are rare. Many patients are treated with chemotherapy, hormonal therapy or a combination of both. Radiotherapy was strongly recommended by Puglisi et al. in elderly with anal involvement. Surgery should be limited to cases with complications such as stenosis or obstruction, or for obtaining a diagnosis [2, 4, 10, 11, 14].

Although survival of patients with BC and GI metastasis is increasing, prognosis is still poor. GI tract metastasis is often associated with extensive disseminated disease. Our patient had progressive bone metastases, peritoneal involvement was seen during laparoscopy and later on a suspect gastric lesion was found. Average survival after diagnosis of GI metastases is 1–2 years [6, 10, 14].

This case report highlights the importance of a high index of clinical suspicion for metastatic disease in a patient with a previous history of breast malignancy, ILC in particular, presenting with new GI symptoms. Clinical, endoscopic and radiological features may be variable, non-specific and misleading. This makes a correct diagnosis difficult and probably causes an underestimation of this pathology. Recognizing that GI metastasis may occur many years after the primary diagnosis of BC is important and demonstrates the need for long-term follow-up. Early and accurate diagnosis is essential for subsequent appropriate treatment. As GI tract involvement is mostly seen with extensive metastatic disease, prognosis is still poor.

## Abbreviations

BC: breast cancer
ILC: invasive lobular carcinoma
GI: gastrointestinal
ER: estrogen receptor
PR: progesterone receptor
HER2-Neu: human epidermal growth factor receptor 2
CA: cancer antigen
CEA: carcinoembryogenic antigen
CT: computerized tomography
MRI: magnetic resonance imaging
